# The evolution, metabolism and functions of the apicoplast

**DOI:** 10.1098/rstb.2009.0273

**Published:** 2010-03-12

**Authors:** Liting Lim, Geoffrey Ian McFadden

**Affiliations:** School of Botany, University of Melbourne, Parkville, Victoria 3010, Australia

**Keywords:** plastid, apicoplast, parasite, apicomplexan, evolution, metabolism

## Abstract

The malaria parasite, *Plasmodium falciparum*, harbours a relict plastid known as the ‘apicoplast’. The discovery of the apicoplast ushered in an exciting new prospect for drug development against the parasite. The eubacterial ancestry of the organelle offers a wealth of opportunities for the development of therapeutic interventions. Morphological, biochemical and bioinformatic studies of the apicoplast have further reinforced its ‘plant-like’ characteristics and potential as a drug target. However, we are still not sure why the apicoplast is essential for the parasite's survival. This review explores the origins and metabolic functions of the apicoplast. In an attempt to decipher the role of the organelle within the parasite we also take a closer look at the transporters decorating the plastid to better understand the metabolic exchanges between the apicoplast and the rest of the parasite cell.

## 1. Introduction

The apicoplast is a vestigial plastid present in most parasites of the Phylum Apicomplexa. The group derives its name from the apical complex, a collection of anterior structures that allow the parasite to invade host cells and establish themselves therein. Apicomplexans are responsible for a wide range of serious diseases of humans, livestock, wild animals and invertebrates and there are an estimated 5000 species of parasites in the group. Human apicomplexan diseases include malaria and toxoplasmosis; babesiosis, theileriosis and coccidiosis are common problems in livestock. Malaria is a major global health problem with 3.3 billion at risk of infection and an estimated 250 million cases per year that resulted in nearly a million deaths in 2006 (WHO world malaria report 2008). Malaria is endemic in tropical areas owing to warm temperatures and high humidity, which are conducive to transmission by the mosquito vectors. Most of the disease burden lies in Africa, where treatment accessibility is the greatest limiting factor. Consequently, malaria is often considered a ‘poor man's disease’ and, unlike most diseases, the malaria problem is becoming worse rather than better.

The current greatest challenge in malaria management is the resistance of parasites to conventional monochemotherapies like chloroquine and sulphadoxine-pyrimethamine. These therapies were cheap and effective but they are losing efficacy worldwide owing to resistance. Artemisinin-based combination therapies are currently the most effective treatment and patients' drug compliance is also reasonable. Although combinatorial therapies are proving to be more effective in fighting malaria, and resistance will hopefully take longer to erode their utility, it is imperative to discover more drug targets to manage the disease before any more drugs lose clinical relevance. Indeed, we need to identify as many new drug leads as possible to expand our repertoire of drugs to use in combination therapies and also to allow rotation to minimize resistance.

The discovery of the apicoplast ushered in an exciting new prospect for drug development against *Plasmodium falciparum*. The eubacterial ancestry of the organelle offers a wealth of opportunities for the development of therapeutic interventions (McFadden *et al*. 1996; McFadden & Roos 1999). Morphological, biochemical and bioinformatic studies of the apicoplast have further reinforced its ‘plant-like’ characteristics and potential as a drug target. However, we are still totally ignorant about why the apicoplast is essential to the parasite's survival (Fichera & Roos 1997; He *et al*. 2001). This review explores the origins and metabolic functions of the apicoplast. In an attempt to decipher the role of the organelle within the parasite we also take a closer look at the transporters decorating the plastid to better understand the metabolic exchanges between the apicoplast and the rest of the parasite cell.

## 2. Origin and evolution

### (a) Where did the apicoplast come from?

The apicoplast is clearly of secondary endosymbiotic origin, which refers to one eukaryote having engulfed and retained another eukaryote with a plastid obtained by primary endosymbiosis of a cyanobacterium-like prokaryote. Secondary endosymbiotic plastids typically have three or four membranes, whereas primary plastids invariably have only two membranes, which are homologous to the two membranes of the Gram-negative ancestral cyanobacterium like endosymbiont. Some observers describe three membranes around the apicoplast but the majority see four, and no one claims to see two, so a secondary endosymbiotic origin is agreed upon. The outermost membrane of the apicoplast is analogous to the phagosomal membrane of the host cell, hence it is also of endosomal origin. The second outermost membrane of the apicoplast originates from the plasma membrane of the engulfed alga cell. The inner pair of membranes is equivalent to the outer and inner envelopes of the chloroplast, which evolved from the cell surface envelope of the engulfed cyanobacterium by the primary host.

What is not agreed however is the nature of the secondary endosymbiont. One school contends that the endosymbiont was a green alga (Kohler *et al*. 1997; Funes *et al*. 2002), whereas another school favours a red algal endosymbiont. In our view, a red algal endosymbiont is now proven beyond much doubt by the discovery of the photosynthetic apicomplexan *Chromera*, which clearly has a plastid derived from a red alga that has the same origins as the apicoplast. A red algal origin for the apicoplast is also part of a much broader hypothesis for secondary endosymbiotic origin of plastids in a large group of protists known as the chromalveolates. The chromalveolate theory proposes that all algae belonging to Chromalveolata possess secondary red plastids acquired by a single common endosymbiotic event, whereby a rhodophyte alga is engulfed by a heterotrophic eukaryotic host (Cavalier-Smith 1999). This supergroup includes Apicomplexa, Dinophyta, Ciliophora, Heterokonts, Haptophyta and Cryptophyta. In support of the chromalveolate hypothesis, structural characteristics of the plastid genome (Blanchard & Hicks 1999) and phylogenetic analyses of the nuclear-encoded plastid-targeted *GAPDH* (Fast *et al*. 2001) and *coxII* (Waller *et al*. 2003*a*) genes reinforced the red algal origin of the apicoplast. Similarly, the apicoplast genome architecture is also more consistent with a red algal ancestry (Blanchard & Hicks 1999; McFadden 2000; Fast *et al*. 2001; Harper & Keeling 2003).

Members of Apicomplexa, including *P. falciparum*, *Toxoplasma gondii*, *Eimeria tenella*, *Babesia bovis* and *Theileria annulata*, and the above-mentioned photosynthetic *Chromera* spp. all harbour an apicoplast. However, the apicoplast is apparently absent from gregarines (Toso & Omoto 2007), colpodellids (Kuvardina *et al*. 2002) and *Cryptosporidium* spp. (Zhu *et al*. 2000) and these members are now presumed to have lost their apicoplasts.

There are no fossil records for apicomplexa but molecular dating estimates the apicomplexan protists to have evolved between 600–800 Ma ago (Douzery *et al*. 2004) and there are fossils of the sister group dinoflagellates that are in excess of 400 million years old. Therefore, it is reasonable to assume that the original free-living apicomplexan parasitized marine invertebrates prior to the establishments in terrestrial vertebrates (McFadden & Waller 1997; Okamoto & McFadden 2008; Obornik *et al*. 2009). The discovery of the free-living and coral-associated *Chromera velia* is apparently a transition form from autotrophic symbiont to parasite and provides a glimpse into the earliest form of the apicomplexan–animal interaction (Moore *et al*. 2008). Ultrastructural examinations and molecular phylogenetic analyses demonstrated this new organism to be the closest known photosynthetic relative to apicomplexan parasites and the common origin of its plastid to the apicoplasts (Moore *et al*. 2008). The autotrophic nature of *C. velia* allows it to be cultivated independently of its coral host, which provides a model to study apicomplexan evolution and what makes the apicoplast essential (Moore *et al*. 2008).

### (b) The apicoplast and the mitochondrion constitute the sticky duo

One striking feature of the apicoplast is its close proximity to the single mitochondrion (van Dooren *et al*. 2006). Early electron micrographs of various species of *Plasmodium* revealed a ‘spherical body’ in intimate association with the single mitochondrion within the parasites (Aikawa 1966; Hepler *et al*. 1966). Initially the ‘spherical body’ was speculated to be a metabolic store for the mitochondrion (Aikawa 1966; Hepler *et al*. 1966), but we now know this body as the apicoplast (McFadden *et al*. 1996; Kohler *et al*. 1997). Indeed the closeness of the two organelles seems attributable to their metabolic dependences (Ralph *et al*. 2004; van Dooren *et al*. 2006) discussed below.

**Figure 1.**
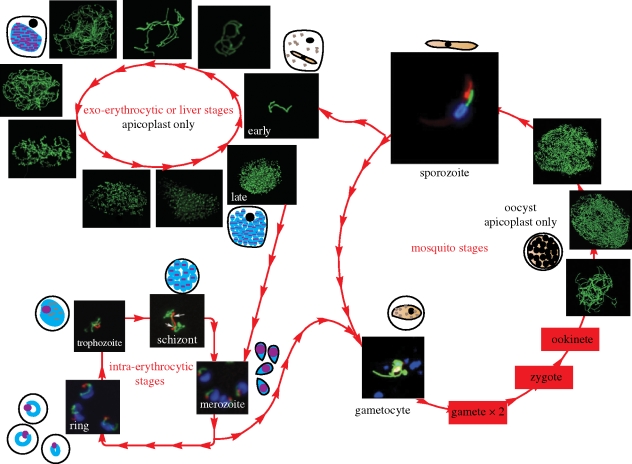
Morphology of the apicoplast throughout the different life stages of *Plasmodium*. Images of the malaria parasite at different life stages are taken from Van Dooren *et al.* (2005), Okamoto *et al.* (2009) and Stanway *et al.* (2009). In the intra-erythrocytic stages and the gametocyte. Green denotes mitochondrion; red denotes apicoplast; blue denotes nucleus. In the sporozoite and other non-erythrocytic stages of the parasite, green denotes apicoplast; red denotes mitochondrion; blue denotes nucleus. (Reproduced with permission from Stanway *et al.* (2009), The Biochemical Society, http://www.biolcell.org).

During its life cycle the *Plasmodium* parasite undergoes three rounds of asexual reproduction: erythrocytic schizogony, sporogony within the oocyst in the mosquito's midgut wall, and exo-erythrocytic schizogony within the liver cell. What happens to the apicoplast during these cell proliferation stages? Genetic manipulation and reporter constructs have facilitated multiple labelling of intracellular compartments in *Plasmodium* (van Dijk *et al*. 1995; Wu *et al*. 1995) allowing the organelle to be observed in live parasites. Throughout the various asexual stages of the parasite, the apicoplast is always in close contact with the mitochondrion (figure 1; van Dooren *et al*. (2005, 2006; Stanway *et al*. 2009). In erythrocytic stages the apicoplast starts out as a relatively simple round structure, elongates, branches extensively and eventually divides such that each daughter cell has a single small apicoplast (van Dooren *et al*. 2005, 2006; Stanway *et al*. 2009). In contrast to the apicoplast in asexual stages, the apicoplast in gametocytes remains simple and unelaborated morphologically but its intimate relationship with the mitochondrion is preserved (figure 1; Okamoto *et al*. 2009; Stanway *et al*. 2009). It is noteworthy that the apicoplast and mitochondrion were only observed in female gametocytes and this is congruent with the maternal inheritance of the organelles (Sinden *et al*. 1976, 1978; Creasey *et al*. 1994; Okamoto *et al*. 2009; Stanway *et al*. 2009).

### (c) Endosymbiotic gene transfer and apicoplast protein import

Compared with the usual photosynthetic plastid genomes, the 35 kb circular apicoplast genome is one of the smallest known to date (Reith & Munholland 1993; Wilson *et al*. 1996; Oudot-Le Secq *et al*. 2007; Rogers *et al*. 2007; Obornik *et al*. 2009). As a consequence of the establishment of an endosymbiont as an organelle, many genes of the endosymbiont have transferred to the host cell nucleus (Martin & Herrmann 1998). Endosymbiotic gene transfer likely minimizes the effects of Muller's ratchet, whereby non-recombining genomes accumulate deleterious mutations. Nuclear primacy probably also serves to provide more subtle gene regulation so that the host cell can manage its endosymbiont better (Martin & Herrmann 1998). In general, the apicoplast genome encodes less than 50 proteins and a great majority are encoded by nuclear genes and the products targeted into the organelles (Wilson *et al*. 1996; McFadden 2000).

The general pathway by which most nuclear-encoded proteins traffic to the apicoplast is mediated by a bipartite leader at the N-terminus of a polypeptide chain (Waller *et al*. 1998, 2000). This leader sequence comprises a signal peptide (SP), which commits the nascent polypeptide chain into the endomembrane system in which the apicoplast is positioned, and a transit peptide (TP), which takes the protein into the plastid (Waller *et al*. 2000). Positive charges at the N-terminus of the TP are essential for faithful apicoplast targeting but the TP lacks any consensus and no secondary structure is evident (Foth *et al*. 2003; Tonkin *et al*. 2006, 2008*a*). Recent work has focused on the machinery in apicoplast membranes that translocates the protein cargo across the apicoplast membranes. Transport across the outermost membrane is the courtesy of the SP. Passage through the next membrane (the periplastid membrane) is now believed to be mediated by an extra set of endosymbiont-derived endoplasmic reticulum-associated degradation complex (Sommer *et al*. 2007; Tonkin *et al*. 2008*b*; Kalanon *et al*. 2009; Spork *et al*. 2009). Since the inner pair of apicoplast membranes is homologous to that of the primary plastids, the translocon of outer envelope of chloroplast (TOC) and translocon of inner envelope of chloroplast (TIC) complexes are postulated to facilitate protein import (van Dooren *et al*. 2001; Tonkin *et al*. 2008*b*). Thus far no TOC components have been identified in apicoplasts, but two TIC components—Tic20 and Tic22—have been described in or associated with the inner apicoplast membrane (van Dooren *et al*. 2008; Kalanon *et al*. 2009).

## 3. What is the function of the apicoplast? metabolic pathways of the organelle

**Figure 2.**
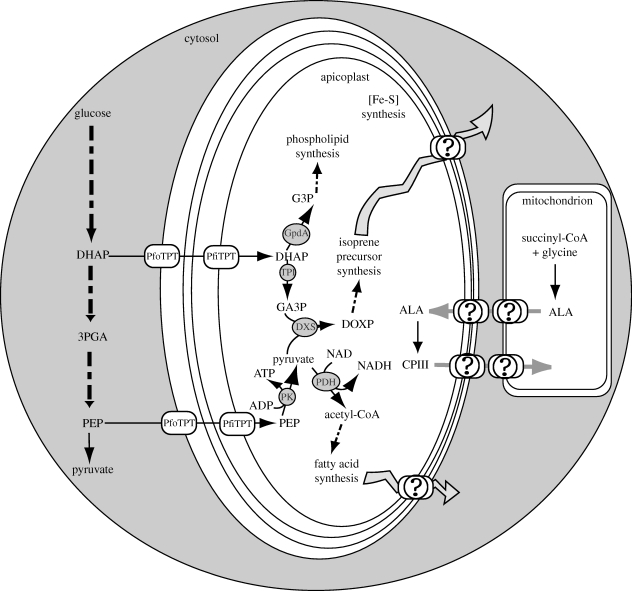
Metabolic map of apicoplast in relation to the mitochondrion in *Plasmodium*. PfoTPT and PfiTPT are the only identified transporters on the apicoplast. They are hypothesized to supply carbon and energy to fuel the metabolic pathways of the organelle. The nature and fates of the substances the apicoplast is predicted to make are unknown, as indicated by the question marks. ALA: aminolaevulinic acid; CPIII: coproporphyrinogen III; DHAP: dihydroxyacetone phosphate; DOXP: 1-deoxy-D-xylulose-5-phosphate; DXS: DOXP synthase; G3P: glycerol-3-phosphate; GA3P: glyceraldehyde-3-phosphate; GpdA: glycerol-3-phosphate dehydrogenase; 3PGA: 3-phosphoglyceric acid; PDH: pyruvate dehydrogenase; PEP: phosphoenolpyruvate; PfiTPT: *P. falciparum* innermost triose phosphate translocator; PfoTPT: *P. falciparum* outermost triose phosphate translocator; PK: pyruvate kinase; and TPI: triose phosphate isomerase.

Since the apicoplast is non-photosynthetic but is essential to the parasite, the plastid community was very intrigued by its function. The small apicoplast genome provided insufficient hints to what the apicoplast is doing besides basic metabolic processes such as DNA replication, transcription and translation (Wilson *et al*. 1996) and attention shifted to the nuclear-encoded apicoplast proteins for functional clues. Because nuclear-encoded apicoplast stromal proteins require a bipartite leader for targeting into the organelle, they are relatively simple to identify from the genome and two bioinformatic tools, PATS and PlasmoAP, are available for predicting *P. falciparum* proteins residing in the apicoplast (Zuegge *et al*. 2001; Foth *et al*. 2003). The former is a neural network-based algorithm while the latter works on a set of rules that identifies putative targeting leaders based on amino acid frequency and distribution. A predicted apicoplast proteome has been assembled using these tools, and putative pathways for the biosyntheses of fatty acids, isoprenoids, iron-sulphur clusters and haem have been mapped out in the apicoplast (figure 2; Ralph *et al*. 2004). These metabolic pathways are essentially the same as those found in bacteria because the apicoplast is of endosymbiotic origin and they are distinct from the pathways found in the mammalian host. It remains to be shown which of these pathways make the apicoplast indispensable.

One of the interesting issues relating to the apicoplast is the unique ‘delayed death’ phenomenon observed of parasites treated with drugs that perturb its basic housekeeping processes (Ramya *et al*. 2007). As expected of its eubacterial ancestry, the machinery that maintains the apicoplast is prokaryotic in origin. Treatment of the malaria parasites with ciprofloxacin, an inhibitor of the bacterial DNA gyrase, and other antibiotics including chloramphenicol, clindamycin, tetracycline and rifampicin resulted in the arrest of growth in the second asexual cycle, while the parasites in the current cell cycle appeared relatively unaffected (Geary *et al*. 1988; McFadden & Roos 1999; Surolia *et al*. 2004; Ramya *et al*. 2007). In contrast, drugs that disrupted the biosyntheses of fatty acids, isoprenoids and haem resulted in relatively rapid death of the parasites (Jomaa *et al*. 1999; Surolia & Surolia 2001; Waller *et al*. 2003*b*; Ramya *et al*. 2007). Together with the observed compromise or loss of apicoplasts in parasites treated with antibiotics, the rapid elimination of parasites with drugs targeting the apicoplast metabolic pathways point towards one or more anabolic products of the plastid being essential for the parasites to establish new infections (Ramya *et al*. 2007). It is generally believed that the metabolic pathways of the apicoplast contribute to lipid production and the modification of lipid-bound proteins (Ralph *et al*. 2004). In this light, the organelle most probably plays a crucial role in the successful establishment of parasite–host interaction and this is likely the formation of a functional parasitophorus vacuole (Ralph *et al*. 2004). Understanding the ‘delayed death’ phenomenon is important in using the apicoplast as a drug target as there are serious clinical outcomes in the rapid clearance of parasites versus the kicking in of drug effectiveness after 48 h of drug administration.

Despite having developed a relatively detailed metabolic map of the apicoplast (Ralph *et al*. 2004), we are still largely ignorant of what metabolic products it actually makes and what is their role in parasites. Experiments to study the roles of the metabolic products are difficult to design and conduct, not least because the parasites are able to scavenge some of the products from the host cells in addition to those made *de novo* complicating the analyses (Bisanz *et al*. 2006). The challenges in resolving the functions of the apicoplast are compounded by its small size and difficulties in isolation, which hamper efforts in biochemical manipulations of the organelle. This following section provides an update on the metabolic pathways housed within the apicoplast in *P. falciparum* to facilitate an appreciation of how each of them contributes to the survival of the parasite. The apicoplast of the malaria parasite is the best characterized among apicomplexan parasites and is described here. We caution however, that not all apicoplast functions are conserved across Apicomplexa.

### (a) Fatty acid synthesis

The type II fatty acid synthesis (FASII) pathway is the best characterized of all the known metabolic pathways the apicoplast houses. Unlike the cytosolic type I pathway found in typical eukaryotes where the fatty acid synthase is a huge multifunctional polypeptide, the FASII pathway in the apicoplast is constituted by separate enzymes (Waller *et al*. 2003*b*; Ralph *et al*. 2004). The conversion of acetyl-CoA to malonyl-CoA by acetyl CoA carboxylase (ACCase) is the first committed step of the pathway. Fatty acid elongation is achieved with rounds of priming the acyl carrier protein (ACP) with a malonyl moiety which adds two carbons to the growing acyl chain in each round by a series of condensation, reduction, dehydration and reduction reactions. The first clue in the discovery of the apicoplast FASII pathway was the identification of nuclear FASII genes whose products are targeted to the apicoplast. Initial drug studies at first seemed to confirm the presence of FASII in blood-stage parasites (Waller *et al*. 1998; Surolia & Surolia 2001; Waller *et al*. 2003*b*) but recent gene deletion studies suggest that apicoplast FASII is only essential in liver stages of the parasite (Yu *et al*. 2008; Vaughan *et al*. 2009). Vaughan *et al*. (2008) successfully deleted β-ketoacyl-ACP synthase I/II (FabB/F) and β-hydroxyacyl-ACP dehydratase (FabZ) from the genome of *P. yoelii* and showed that the enzymes were only critical to late liver-stage parasites while parasites in blood and mosquito stages were unaffected. Deletion of enoyl-ACP reductase (FabI) in *P. falciparum* also did not affect parasite replication in blood stage (Vaughan *et al*. 2009). Yu *et al*. (2008) further supported this notion in another study where the FabI-deficient *P. berghei* parasites compromised with infectivity *in vivo* and often failed to complete liver-stage development while growth in blood stages was unaffected (Yu *et al*. 2008). Considering that liver-stage schizogony gives rise to thousands of fold more merozoites than blood-stage schizogony (Prudencio *et al*. 2006), the apparent necessity of the apicoplast FASII pathway for liver-stage replication but not blood-stage division suggests that the apicoplast probably provides one or more metabolic products with levels that cannot be met by scavenging at late liver stage but the amount required in blood stage does not need to be supplemented by the plastid (Yu *et al*. 2008; Vaughan *et al*. 2009).

Analysing the roles of various metabolic pathways like FASII throughout the complex life cycle of an apicomplexan parasite proves to be very important and insightful. Relying on studies on parasites at a single stage to map the contributions of pathways can indeed be misleading. It was long thought that FASII was important to blood-stage parasites because of the observed growth inhibition of triclosan-treated asexual blood-stage parasites (Surolia & Surolia 2001). Moreover, triclosan is known to specifically inhibit the bacterial FabI (Stewart *et al*. 1999; Heath *et al*. 2002). Nevertheless, Yu *et al*. (2008) have demonstrated from binding studies that the *Plasmodium* FabI and bacterial FabI are different as the former is not targeted by triclosan. In addition, triclosan was found to affect the growth of *T. parva* even though the apicoplast of the parasite appears to lack FASII components, including FabI (Gardner *et al*. 2005; Lizundia *et al*. 2009). Control studies on non-infected host cells demonstrated that triclosan indeed has non-parasite-specific off-target effects (Lizundia *et al*. 2009). Therefore, caution should also be exercised when extrapolating findings from other organisms.

If FASII is redundant to blood-stage parasites, the presence of FASII enzymes such as ACCase (D. Goodman 2009, personal communication), pyruvate dehydrogenase (PDH) complex (Foth *et al*. 2005) and ACP in the *Plasmodium* apicoplast is somewhat anomalous (Waller *et al*. 2000; van Dooren *et al*. 2002; Foth *et al*. 2005). The gene knock out data demonstrates that blood-stage parasites survive when FabI is absent (Yu *et al*. 2008) so why are components of the pathway expressed at all? One explanation is that FASII at blood stage could simply be supplying lipoic acid, a potent antioxidant, to protect the parasite against oxidative insults—ingestion of haemoglobin from the erythrocyte subjects the parasite to increased oxidative stress (Toler 2005). The lipoic acid generated *de novo* can also be used as a cofactor for the mitochondrial α-keto acid dehydrogenase (van Dooren *et al*. 2006). However, exogenous addition of lipoic acid to triclosan-treated parasites did not rescue them (Ramya *et al*. 2007) but this could be owing to the unexpected toxicity of triclosan as mentioned previously (Lizundia *et al*. 2009). The presence of ACCase, PDH and ACP at blood stage may, alternatively, be attributed to the lack of mechanism for the downregulation of the genes or turnover of the proteins. We await further studies to resolve the conundrum.

### (b) Isoprenoid synthesis

Isoprenoids are made up of repeated isopentenyl pyrophosphate (IPP) or dimethylallyl diphosphate (DMAPP) units. They are prosthetic groups on many enzymes and also precursors to ubiquinones and dolichols, which are involved in electron transport and the formation of glycoproteins, respectively. Similar to bacteria and chloroplasts, the apicoplast harbours the non-mevalonate/2-C-methyl-d-erythritol 4-phosphate (MEP)/1-deoxy-d-xylulose-5-phosphate (DOXP) pathway for isoprenoid synthesis (Jomaa *et al*. 1999; Ralph *et al*. 2004). Like FASII, the apicoplast is the sole site of isoprenoid precursor synthesis in the *Plasmodium* parasite. A striking difference between the DOXP pathway, which has only relatively recently been elucidated, and the canonical mevalonate pathway found in the eukaryotic cytoplasm is the starting substrates. The former uses pyruvate and glyceraldehyde-3-phosphate to produce IPP and DMAPP, while the latter starts with the condensation of acetyl-CoA and acetoacetyl-CoA to form 3-hydroxy-3-methylglutaryl-CoA (HMG-CoA), which is subsequently reduced to mevalonate for the pathway. This difference means there is essentially no commonality between the two pathways making DOXP an ideal target for antibacterials and antimalarials.

Evidence of active isoprenoid synthesis in the *Plasmodium* apicoplast stems from the presence of transcripts of DOXP reductoisomerase (IspC) and DOXP synthase (Jomaa *et al*. 1999) and the detection of several metabolic intermediates of the DOXP pathway in asynchronous blood-stage cultures (Cassera *et al*. 2004). In contrast to FASII, the DOXP pathway for isoprenoid biosynthesis in the apicoplast appears to be essential to blood-stage parasites as the antibiotic fosmidomycin, an inhibitor of IspC (Kuzuyama *et al*. 1998), is effective in managing the clinical symptoms of malaria that are associated with the intra-erythrocytic phase of parasites (Jomaa *et al*. 1999). It is, however, noteworthy that fosmidomycin is poorly effective against the coccidians *E. tenella* and *T. gondii* despite the presence of the isoprenoid genes in these parasites (Clastre *et al*. 2007). Differences in the nature of host cell type (Clastre *et al*. 2007) and the IspC orthologues may underlie the differences in drug sensitivity.

Interestingly, the effect of fosmidomycin on levels of DOXP pathway intermediates and metabolites was found to be most prominent in ring stages followed by schizonts among the different blood-stage forms of *P. falciparum* (Cassera *et al*. 2004). This probably forms the rationale for using fosmidomycin in combination with another drug for better disease control (Borrmann *et al*. 2004, 2005). Within the apicoplast, DMAPP is likely used in the isopentenylation of tRNAs as four apicoplast-encoded tRNAs (trnW^CCA^, trnC^GCA^, trnL^UAA^ and trnY^GUA^) are suitable candidates for modification (Ralph *et al*. 2004). Besides apicoplast translation, the DOXP pathway also potentially provides precursors for the synthesis of ubiquinones for the electron transfer system in the mitochondrion, protein prenylation and the formation of dolichols for glycosylphosphatidyl inositol (GPI)-anchors on membrane proteins found on many *Plasmodium* surface proteins (Naik *et al*. 2000; Ralph *et al*. 2004). A role for the apicoplast supply of isoprene tails for mitochondrial ubiquinone is supported by the decrease in ubiquinone content in fosmidomycin-treated rings (Cassera *et al*. 2004). Paradoxically, inhibition of isoprenoid synthesis in rings impacted upon dolichol level the greatest in rings but had no significant effect on trophozoites (Cassera *et al*. 2004) despite radiolabelling studies demonstrating that GPI synthesis mainly takes place in the latter (Naik *et al*. 2000).

### (c) Iron-sulphur cluster synthesis

Cellular requirements for iron-sulphur clusters are typically met by the *de novo* [Fe-S] cluster synthesis in the mitochondrion. However, the proteome of the apicoplast includes proteins such as ferredoxin (Fd), LipA, IspG, IspH and MiaB, all of which require [Fe-S] clusters, but it seems unlikely that [Fe-S] complex prosthetic groups would be imported from the mitochondrion across the secretory system in which the plastid resides (Seeber 2002; Ralph *et al*. 2004). Moreover, nuclear-encoded [Fe-S] containing proteins are almost certainly imported into the apicoplast in an unfolded state (van Dooren *et al*. 2002; Tonkin *et al*. 2008*b*) and should thus be in the apo-form while in transit (Seeber 2002). Searches of the apicoplast proteome identify various iron-sulphur cluster biosynthetic enzymes including SufB or Orf470 encoded by the apicoplast genome and NifU, SufA, SufC, SufD and SufS in the parasite's nuclear genome (Ellis *et al*. 2001; Seeber 2002; Ralph *et al*. 2004). Although none of the [Fe-S] cluster pathway component has yet been shown to be essential for the maturation of the above-mentioned apicoplast proteins, their roles in other essential processes make it likely that this metabolism is also indispensable. Surprisingly, there is a general lack of drugs known to inhibit this pathway, but the parasite's retention of ferredoxin-NADP^+^ reductase (FNR)/ferredoxin (Fd) redox system for function suggests that perturbation of redox poise by pharmacological interference should kill the parasites (see later section on ‘Powering the apicoplast’).

### (d) Haem synthesis

Like [Fe-S] clusters, haem is an important prosthetic group on many proteins such as cytochromes. Malaria parasites are literally drowning in haem released from the digestion of the haemoglobin they phagocytose from the host cell. Indeed, this haem has to be neutralized to prevent parasite cell damage and the organism does not appear to have evolved a mechanism to access the haem from haemoglobin. Instead, haem from the degradation of haemoglobin in the food vacuole is polymerized to the non-toxic hemozoin crystal (Sullivan *et al*. 1996) and the parasite has a *de novo* haem synthesis pathway. In addition, the parasite also undergoes extra-erythrocytic stages and hence will need the ability to meet its own haem requirements. The haem biosynthetic pathway is essential (Surolia & Padmanaban 1992; Ramya *et al*. 2007) and unusual as components constituting a complete pathway are shared between the apicoplast and the mitochondrion, and haem intermediates are hypothesized to shuttle between the two compartments and possibly the cytosol (Ralph *et al*. 2004; van Dooren *et al*. 2006). The acquisition of a secondary endosymbiont probably gave rise to the presence of two haem biosynthetic pathways in the organism and the unusual hybrid pathway likely emerged as components were lost to eliminate redundancy (Ralph *et al*. 2004).

The unique conjoined haem pathway in *Plasmodium* is one of the more bizarre examples of evolutionary rationalization of redundancy. When the host originally procured a secondary endosymbiont it would appear to have found itself in possession of two separate haem pathways: a canonical Shemin pathway partitioned across the mitochondrion and the cytosol, plus an additional, self-contained pathway of cyanobacterial type in the endosymbiont (apicoplast). Rationalization involved loss of the cytosolic components of the Shemin-type pathway and substitution of the equivalent steps from the apicoplast pathway to create a hybrid pathway that runs cooperatively between the two endosymbiont organelles. Determining the localizations of the various enzymes of the pathway is fundamental to appreciating how the terminal product haem is used in the parasite. δ-Aminolaevulinic acid (ALA) synthase (PfALAS) localizes to the mitochondrion (Varadharajan *et al*. 2002) and kick-starts the pathway to provide ALA. The pathway then shifts to the apicoplast as δ-aminolaevulinate dehydratase (PfALAD or HemB (Sato & Wilson 2002; van Dooren *et al*. 2002), porphobilinogen deaminase (PfPBGD or HemC; Sato *et al*. 2004) and uroporphyrinogen III decarboxylase (UROD or HemE; Nagaraj *et al*. 2009) localize to the plastid (Sato *et al*. 2004). Early reconstructions of the haem pathway were missing HemD (van Dooren *et al*. 2006), but this conundrum was resolved when PfPBGD was also found to encompass the function of uroporphyrinogen III synthase (UROS or HemD; Nagaraj *et al*. 2008). It is now imperative to confirm the localization of coproporphyrinogen oxidase (PfCPO or HemF) and protoporphyrinogen oxidase (PfPPO or HemG) to better examine the translocation machinery that may be required on the apicoplast membranes for the transmembrane shuttling of coproporphyrinogen III or the subsequent metabolites. The terminal enzyme catalysing the insertion of the ferrous iron into protoporphyrin IX, ferrochelatase (HemH), is localized to the mitochondrion (van Dooren *et al*. 2006) despite an earlier disputable immunofluorescence assay demonstrating its apicoplast localization (Varadharajan *et al*. 2004).

Localizations of the above were done in the intra-erythrocytic stage of the malaria parasite. Surprisingly, host ALAD was found to be imported into the cytosol of the parasite (Bonday *et al*. 2000). In view of the reduced catalytic efficiencies observed of several of the *Plasmodium* enzymes (PfALAD, PfPBGD and PfUROD) compared with the host orthologues, Nagaraj *et al*. (2008) suggested that that the import of host enzymes might serve to compensate the parasite's *de novo* haem biosynthesis. It would appear strange for the parasite to back up its system as proposed but remains a possibility.

## 4. The chloroplast in darkness: carbon source of the apicoplast

Apicoplast metabolic pathways involving the biosyntheses of fatty acids, isoprenoids, iron-sulphur clusters and haem must be driven by sources of carbon and energy. In the absence of photosynthetic drive to fix carbon, generate ATP and create reducing power, the apicoplast needs to import these components to drive its anabolism. We like to model the apicoplast on non-photosynthetic plastids of plants such as leucoplasts, which also lack the ability to fix carbon and must be ‘fed’ by other parts of the plant. Non-photosynthetic plastids import fuel using specific metabolite transporters on the inner envelope of the plastid known as plastidic phosphate translocators (pPTs). There are four classes of pPTs: triose phosphate/phosphate transporters (TPT), phosphoenolpyruvate phosphate/phosphate transporters (PPT), glucose 6-phosphate/phosphate transporter (GPTs) and xylulose 5-phosphate/phosphate transporter (XPTs; Fischer & Weber 2002). pPTs function as antiporters, where a sugar phosphate is translocated in exchange for an inorganic phosphate (Fischer *et al*. 1997; Fischer & Weber 2002). The TPT, which exports triose phosphates from the illuminated plastid, is the major transporter in photosynthetic plastids as the carbon fluxes it controls affects the rates of intraplastid starch biosynthesis and mobilization and sucrose biosynthesis occurring in the cytosol (Schneider *et al*. 2002).

Generally, the main sources of carbon for making fatty acids in plastids are acetate from the hydrolysis of mitochondrial acetyl-CoA, the end product of glycolysis pyruvate and glycolytic intermediates like glucose 6-phosphate (G6P), phosphoenolpyruvate (PEP), triose phosphates (glyceraldehyde 3-phoshate; GA3P; dihydroxyacetone; DHAP) and malate (Neuhaus & Emes 2000; Rawsthorne 2002). The relative contribution of each precursor to the pathway is specific to plastid types, tissue types and developmental stages of the cells (Qi *et al*. 1994; Neuhaus & Emes 2000). Acetate is probably small enough to enter the plastid via diffusion (Neuhaus & Emes 2000). The GPT (Kammerer *et al*. 1998), PPT (Fischer *et al*. 1997) and TPT (Fischer *et al*. 1994) are involved in the import of G6P, PEP and triose phosphates, respectively, while malate is taken up via the 2-oxoglutarate/malate translocator (Weber *et al*. 1995). Pyruvate may enter from the cytosol through a pyruvate transporter that is yet to be characterized or it can also be produced from PEP by the plastidic pyruvate kinase via the intraplastid glycolytic pathway (Neuhaus & Emes 2000).

Plastidic phosphate translocators are ancient and the secondary endosymbiont ancestor of the apicoplast likely had at least one, and perhaps a small suite of pPTs to manage its relationship with the host. What can we deduce about the current non-photosynthetic status of the apicoplast and its pPTs? One way to double guess the mechanisms that power apicoplasts is to map out what is required and review the inventory of carbon and energy-metabolizing enzymes in the apicoplast proteome. Like most non-photosynthetic plastids, the apicoplast lacks hexose- or pentose-processing components (Qi *et al*. 1994; Ralph *et al*. 2004). Therefore, the apicomplexan plastid was hypothesized to import C3 compounds like triose phosphates and PEP from the cytosol to fuel its metabolic pathways (Ralph *et al*. 2004).

Two pPT homologues (PfiTPT and PfoTPT) were identified in the *P. falciparum*'s genome (Gardner *et al*. 2002) and it has been suggested that they are likely to transport triose phosphates and PEP into the apicoplast to sustain carbon metabolism (Ralph *et al*. 2004; Mullin *et al*. 2006). PfiTPT has a bipartite leader and is localized in an apicoplast membrane, probably the innermost apicoplast membrane by analogy with plant plastid pPTs and the fact that the N-terminus is processed like stromal apicoplast proteins (Mullin *et al*. 2006). PfoTPT, on the other hand, lacks a leader and clearly resides in the outermost membrane of the apicoplast (Mullin *et al*. 2006) as evidenced by its accessibility to protease cleavage and antibodies to the termini in free but intact apicoplasts (Mullin *et al*. 2006). Considering the lack of other candidates in the inner pair of apicoplast membranes, these two transporters PfoTPT and PfiTPT are postulated to work in tandem to facilitate the import of triose phosphates and PEP to channel the substrates into the FASII and isoprenoid biosynthesis pathways but how such substrates cross the middle two apicoplast membranes remains unclear (Mullin *et al*. 2006; Lim *et al*. 2009). It is noteworthy that pPTs are not required to cross the outer plastid membrane in plants and low specificity pores such as OEP21 facilitate passage (Bolter *et al*. 1999), and such porin-like protein channels may occur in the intermediate apicoplast membranes, but none have been identified.

Besides some typical plastid metabolisms in the apicoplast, the presence of glycolytic components in the apicoplast stroma is another endorsement of an engulfed organism within the parasite. However, it is interesting to question the role the triose phosphate isomerase (TPI) plays in the apicoplast. If PfiTPT and PfoTPT were indeed involved in the import of triose phosphates into the apicoplast, the affinities of the transporters for GA3P and DHAP probably differ to favour the translocation of the latter since the presence of TPI validates its relevance and it probably converts imported DHAP to GA3P for reaction with pyruvate to form DOXP for the isoprenoid biosynthesis pathway (Ralph *et al*. 2004). The proposed flexibilities of PfiTPT and PfoTPT in transporting DHAP, GA3P and PEP should not be surprising since the plant pPTs do not seem to have very restrictive substrate preferences (Fischer *et al*. 1997).

## 5. Apicoplast carbon sources across apicomplexan parasites: bioinformatic survey of parasite ppt orthologues

Apicoplasts of the different apicomplexan parasites perform different metabolic activities. How does their complement of carbon transporters reflect these differing requirements? Previous investigation of *T. gondii* identified a single pPT homologue (named TgAPT1; apicoplast phosphate transporter 1) and localization studies were interpreted as showing that TgAPT1 resided in multiple apicoplast membranes, though a mechanism for such an unusual disposition of a single protein was not afforded (Karnataki *et al*. 2007*a*). TgAPT1 is probably the homologue of PfoTPT and *T. gondii* appears to lack a PfiTPT homologue. What pPTs occur in other apicoplasts? Table 1 shows the pPT orthologues in the various *Plasmodium* species, *T. gondii*, *B. bovis*, *T. parva* and *T. annulata*. Hits were filtered with an *e*-value of not more than 0.01. The orthologues were further selected based on the number of transmembrane domains predicted by TMHMM, the presence of a putative TPT signature which could be a substrate-binding site (Mullin *et al*. 2006), and the general conservation of the sequences with AtTPT.

**Table 1.** Plastidic phosphate translocator orthologues in various apicomplexan parasites, including *Plasmodium* spp., *T. gondii*, *B. bovis*, *T. parva* and *T. annulata*.

organism	GenBank accession ID	number of transmembrane domains predicted	leader prediction
*P. falciparum*	XP_001351641	9	no
*P. falciparum*	XP_001351856	7	yes
*P. knowlesi*	XP_002259733	9	no
*P. knowlesi*	XP_002259508	7	yes
*P. vivax*	XP_001613255	7	no
*P. vivax*	XP_001613659	7	yes
*P. berghei*	XP_677571	8	no
*P. berghei*	XP_677003	6	yes
*P. chabaudi chabaudi*	XP_745978	8	no
*T. gondii*	ABU49222	6	no
*B. bovis*	ABC25608	10	no
*B. bovis*	XP_001609145	8	no
*B. bovis* (BbTPT3)	XP_001610919	5	yes
*B. bovis*	XP_001609146	10	no
*T. annulata*	XP_955232	8	no
*T. parva*	XP_763564	8	no

In general, except for *P. chabaudi chabaudi* whose sequencing is not complete, all the other *Plasmodium* spp. have two copies of pPT each, with one of them having a long N-terminal extension. By comparison with the situation in *P. falciparum* we assume that the leader-bearing copies likely reside within the innermost apicoplast membrane, whereas those without leaders would be expected to be lodged in the outermost membrane (Mullin *et al*. 2006; Tonkin *et al*. 2008*b*; Lim *et al*. 2009). In contrast, *T. gondii* and the two *Theileria* species only have a single pPT each. The *Theileria* pPT likely channels starting materials for the apicoplast IPP biosynthesis as the parasites lack a FASII pathway (Lizundia *et al*. 2009). Interestingly, *B. bovis* appears to have four copies of pPT in its genome with one perhaps bearing a bipartite leader (BbTPT3). It is tantalizing to speculate that each pPT in *B. bovis* is responsible for the transport of sugar phosphates across a particular membrane of the apicoplast. On the other hand, it remains to be established if the pPTs in *Plasmodium* spp., *Toxoplasma* and *Theileria* do indeed span over multiple membranes as has been argued for *T. gondii* or whether other, as yet unidentified proteins like OEP21 on the outer envelope of chloroplast (Bolter *et al*. 1999), which transports anions, exist in the parasites to facilitate the transport of charged substrates like sugar phosphates across the membranes of the apicoplast.

Intriguingly, genome mining also detected distant pPT homologues in *Cryptosporidium* spp. despite the fact that the apicoplast has been lost in this family of apicomplexan parasites (Zhu *et al*. 2000). pPTs are part of a large family of drug metabolite transporters that have varied roles in eukaryotic cells and localize to several different membranes (Martin & Herrmann 1998; Weber *et al*. 2006). Further experimental work will hopefully clarify the origins and current functions of pPTs in apicoplasts.

## 6. Powering the apicoplast: addressing sources of reducing power and atp

Like all plastids, the apicoplast needs ATP and reducing equivalents to power its metabolic pathways like FASII. In the absence of photosynthesis and any plastidic ATP/ADP transporter, the apicoplast has to generate ATP somehow. In the malaria parasite, the conversion of imported PEP to pyruvate for the FASII or DOXP pathway seems to be the sole source of ATP for the apicoplast (Ralph *et al*. 2004). The reaction is catalysed by the plastidic pyruvate kinase (pPK), which is phylogenetically distinct from cytosolic pyruvate kinases and is demonstrated to be an apicoplast-resident enzyme (pPK; L. Lim, N. J. Patron & G. I. McFadden, unpublished data; Saito *et al*. 2008; Maeda *et al*. 2009).

To cope with some of the demands for reductants in the apicoplast, the organelle harbours a plant-type ferredoxin-NADP^+^ reductase (FNR)/ferredoxin (Fd) redox system, which works in a manner similar to the non-photosynthetic FNR/Fd systems (Rohrich *et al*. 2005; Seeber *et al*. 2005). In photosynthetic plastids, Fd receives electrons from photosystem I and the reduced Fd, in turn, is used by FNR to produce NADPH from NADP^+^ for the Calvin cycle. In non-photosynthetic plastids, the ferredoxin redox system operates in the reverse direction. FNR catalyses electron transfer from NADPH to Fd, which then acts as a reductant for various reactions (Rohrich *et al*. 2005). In the apicoplast we know of at least three components that require reduced Fd: lipoic acid synthase (LipA), which provides the potent antioxidant lipoic acid to the E2 subunit of the PDH (Foth *et al*. 2005); and NifU, a protein that provides a scaffold for [Fe-S] to assemble on during [Fe-S] cluster synthesis; and MiaB, which is probably involved in the modification of tRNAs for apicoplast translation (Ralph *et al*. 2004; Seeber *et al*. 2005). Reactions catalysed by the isoprenoid biosynthetic enzymes, GcpE (IspG) and LytB (IspH), also require reduced Fd. In fact, the FNR/Fd system has been demonstrated to be a functional electron shuttle system for IspH, which is the terminal enzyme in the DOXP pathway catalysing the simultaneous production of IPP and DMAPP (Rohrich *et al*. 2005). There is thus a substantial requirement to generate reduced Fd, so where does the NADPH that delivers the electrons to Fd come from?

In plants the pentose phosphate pathway can generate NADPH, but no pentose phosphate pathway is apparent in the apicoplast of *P. falciparum* (Ralph *et al*. 2004). Another source of NADPH in plant plastids is part of a plastid glycolytic pathway for the conversion of GA3P to 1,3-diphosphoglycerate (1,3-DPGA) by GAPDH but again this option appears to be lacking in apicoplasts (Ralph *et al*. 2004). At this stage, our best guess for a source of reduced cofactor in the apicoplast is to invoke a reverse direction of the classic redox shuttle that exports reducing power from photosynthetic plastids (Fleige *et al*. 2007). A reverse triose phosphate/3-phosphoglycerate (3-PGA) shuttle has been proposed to transfer triose phosphates from the cytosol to the apicoplast in exchange for 3-PGA, which effectively results in the generation of an ATP and NADPH in the apicoplast by the action of the plastid-localized phosphoglycerate kinase II and GAPDH, respectively (Ralph *et al*. 2004; Fleige *et al*. 2007). Although GAPDH is localized to the apicoplast in *T. gondii* (Fleige *et al*. 2007), this enzyme is not clearly localized to the apicoplasts of other parasites (table 1).

Alternatively, if NADH can be substituted as a reductant for NADPH in the apicoplast of the malaria parasite, the decarboxylation of pyruvate to acetyl-CoA with the generation of NADH from NAD^+^ by the PDH complex offers a potential source of reductant for the organelle. This will, however, cause a build-up of acetyl-CoA which could limit the efficiency of the FASII pathway. The parasite would then have to evolve a mechanism to overcome this, perhaps by extruding the excess acetyl-CoA from the organelle. If acetyl-CoA were exported from the apicoplast, it would presumably need transporters. An attractive, but purely speculative, scenario would be to export surplus acetyl-CoA to the mitochondrion, which lacks a PDH to generate acetyl-CoA for its TCA cycle (Foth *et al*. 2005). Importing PEP, converting it to pyruvate, then converting the pyruvate to acetyl-CoA that was shuttled to the mitochondrion would provide a means to resolve the ATP and NADH deficits in the apicoplast as well as the presumed acetyl-CoA deficit in the mitochondrion, but the transport of acetyl-CoA across all those membranes remains a hurdle for this postulation.

Together with the uniqueness of the plant-type FNR/Fd redox system in the host cell, its involvement in a range of pathways in the apicoplast makes it an attractive drug target (Seeber *et al*. 2005). Probably, the system is also ready for rational drug design since abundant structural information of the individual FNR and Fd in cyanobacteria and plants are available (Serre *et al*. 1996; Binda *et al*. 1998; Morales *et al*. 1999). Since the Fd/FNR redox system is involved in intra-plastid [Fe-S] cluster synthesis, its importance is further implicated in the insertion of [Fe-S] clusters in several apicoplast enzymes (Ralph *et al*. 2004; Seeber *et al*. 2005). The functional significance of the redox pair has also been unequivocally demonstrated by the elucidation of the molecular interaction of Fd and FNR in *P. falciparum* recently (Kimata-Ariga *et al*. 2007).

## 7. Intracellular role of the apicoplast: unravelling the elusive permeome of the organelle

A potential way to work out the role of the apicoplast within the parasite is to find out what goes in and out of the organelle. By focusing on the gatekeepers—the proteins residing in the apicoplast membranes—we can hope to get a snapshot of exchange between the two partners. The first apicoplast membrane proteins to be identified were PfiTPT and PfoTPT (Mullin *et al*. 2006), and several more membrane proteins have now been uncovered in the apicoplast of *T. gondii*, namely the pPT orthologue TgAPT1 (Karnataki *et al*. 2007*a*), a membrane protease TgFtsH1 (Karnataki *et al*. 2007*b*), a thioredoxin-like protein ATrx1 (DeRocher *et al*. 2008), and apicoplast protein translocation component TgTic20 (van Dooren *et al*. 2008), Tic22 (Kalanon *et al*. 2009) and Der1 (Kalanon *et al*. 2009; Spork *et al*. 2009). While we have preconceived notions of the functions of TgAPT1, TgTic20, Tic22 and Der1, the roles of TgFtsH1 and ATrx1 are less obvious. With the exception of ATrx1, it appears that corresponding homologues also exist in the genome of *P. falciparum* and it seems likely that the apicoplast membrane complement will be comparable (Karnataki *et al*. 2007*a*,*b*; DeRocher *et al*. 2008; Lim *et al*. 2009).

Targeting requirements for apicoplast proteins are reasonably well understood for *Plasmodium* and *Toxoplasma*, these being to most tractable systems experimentally. Initially it was thought that apicoplast proteins required a bipartite N-terminal leader for targeting but PfoTPT, which lacks a leader and targets to the outer apicoplast membrane introduced a new paradigm. Bioinformatic searches against the genomes of *P. falciparum* and *T. gondii* for potential apicoplast transporters with a bipartite leader were initially limited to PfiTPT (Ralph *et al*. 2004). As more membrane components are revealed in the apicoplasts of *Plasmodium* and *Toxoplasma*, a new apicoplast-targeting pathway independent of an N-terminal bipartite leader has emerged (Lim *et al*. 2009). In TgAPT1, TgFtsH1 and ATrx1, a signal anchor or transmembrane domain appears to the common requirement for targeting to the plastid (Karnataki *et al*. 2007*a*,*b*; DeRocher *et al*. 2008; Lim *et al*. 2009). The recessed hydrophobic patch supposedly commits each of the proteins into the endomembrane system in which the apicoplast is positioned. Beyond the signal anchors, the three unrelated proteins do not appear to share any common motif. The targeting requirement is also consistent in *P. falciparum* where the first transmembrane domain of the leaderless PfoTPT is sufficient to direct the protein to the ER (L. Lim & G. I. McFadden 2009, unpublished data). It remains to be confirmed if an alternate degenerate motif exists for apicoplast targeting since varying lengths of ATrx1, TgFtsH1 and TgAPT1 appear to be required for targeting to the plastid (Karnataki *et al*. 2007*a*,*b*; DeRocher *et al*. 2008). If we can determine what motifs target proteins to the apicoplast membranes, we will expand our knowledge of the apicoplast permeome. Doubtlessly, more candidate proteins are necessary to be used for sequence comparison, especially if the new motif is relatively obscure like the vacuolar transport signal (VTS) or *Plasmodium* export element (PEXEL) for trafficking nuclear-encoded proteins beyond the parasitophorus vacuole of *Plasmodium* (Hiller *et al*. 2004; Marti *et al*. 2004).

Until the apicoplast proteome is unravelled biochemically, one model for identifying proteins potentially residing in the apicoplast membranes is by comparison with plant plastids. One bulk communication of metabolites between the chloroplast with the rest of the plant cell is the transfer of lipids into and out of the plastid for intra- and extra-plastidic membrane biogeneses (Benning *et al*. 2006). Most glycerolipids made in the ER require fatty acids derived from the *de novo* synthesis in chloroplasts, although exogenous supply can also be used intracellularly (Ohlrogge & Browse 1995; Koo *et al*. 2005; Benning *et al*. 2006). This scenario is reminiscent of lipid metabolism in the parasite (Bisanz *et al*. 2006). Since the apicoplast is the sole site of lipid biosynthesis in the parasite, it is likely to export lipids. In chloroplasts, lipid fluxes between the ER and plastid are facilitated by plastid-associated microsomes (PLAMs; Benning *et al*. 2006). A few proteins associated with ER–plastid lipid transfer such as the ATP-binding cassette (ABC) transporter TGD1 (Xu *et al*. 2005), and vesicular lipid trafficking from the inner envelope membrane to the thylakoid-like VIPP1 (Kroll *et al*. 2001) are providing the first sketch of lipid routes in the chloroplast. Whereas insights into the components involved in lipid flow into the plastid are progressing well, those facilitating the export of fatty acids from the chloroplast remain somewhat elusive (Benning *et al*. 2006) although another ABC transporter in *Arabidopsis* has been implicated in the export of wax precursors (Pighin *et al*. 2004). Nonetheless, we are optimistic that breakthroughs made by our plant colleagues will help shed light on the mechanism of fatty acid flow to and from the apicoplast.

The apicoplast is also likely to export the IPPs and DMAPPs as well as fatty acids, but the molecular machinery to mediate the process is completely unknown, even in plant plastids. The situation is similar for transmembrane iron transport in iron-sulphur biosynthesis within the plastids (Briat *et al*. 2007). Several apicoplast proteins like ferredoxin bear iron-sulphur clusters and they are most likely imported into the apicoplast in the apo-form (Seeber 2002). Presence of the [Fe-S] biosynthesis in the apicoplast negates the need to import the prosthetic groups for the apicoplast proteins but the suite of *Suf* proteins in the organelle would not be able to function without a supply of iron and remains a complete mystery until candidate transporters are identified, or the form in which the iron enters the organelle is known.

The apicoplast membranes should also harbour components that mediate the exchange of haem intermediates with mitochondrion (figure 2; van Dooren *et al*. 2006). In *P. falciparum*, transporters are presumably necessary to transport δ-aminolaevulinic acid out of the mitochondrion into the plastid and to facilitate the entry of coproporphyrinogen III from the apicoplast into the cytosol or mitochondrion (van Dooren *et al*. 2006; Nagaraj *et al*. 2009). Considering the intimate association of the two organelles (Bannister *et al*. 2000; van Dooren *et al*. 2006; Okamoto *et al*. 2009), a membranous continuum to mediate the metabolite exchanges between the two cannot be discounted but there is no evolutionary precedent for such a continuum. To date, several mitochondrial porphyrin transporters are identified, including the PBR and ABCB6 candidate transporters on the outer mitochondrial membrane (Verma *et al*. 1987; Krishnamurthy *et al*. 2006) and ANT on the inner membrane (Azuma *et al*. 2008). However, no plastid haem-associated transporters are known, but they probably exist (van Dooren *et al*. 2006). On the other hand, comparative studies with the bacterial haem transport systems might shed light on apicoplast mechanisms (Tong & Guo 2009).

## 8. Concluding remarks

Much attention has been centred on the apicoplast since its identification 13 years ago. This review has provided glimpses into the various efforts to understand the evolutionary origin and metabolic functions of the plastid. This interesting four-membraned organelle is not the only feature that makes the apicomplexan parasite ‘plant-like’. Recently, the presence of AP2 transcription factors (Balaji *et al*. 2005) and the carotenoid biosynthesis pathway (Tonhosolo *et al*. 2009) have recalled the endosymbiotic history of the parasite.

As we gain insights of pathways unique to the parasite, it is imperative to follow up the discovery of new drug targets and forge on to clinical evaluations. There is no doubt that transporters on the apicoplasts should be of high priority. The apicoplast gatekeepers limit the downstream metabolism and should be ideal targets for intervention. Our challenge is to explore and understand how the organelle's permeome works so that we can use this knowledge to help the unfortunate sufferers of apicomplexan diseases.
